# Supplementary material for: The Metabolic Benefits of Menopausal Hormone Therapy Are Not Mediated by Improved Nutritional Habits. The OsteoLaus Cohort
Source: Nutrients. 2019 Aug 16;11(8):1930. doi: 10.3390/nu11081930 (PMC6722637; doi:10.3390/nu11081930)
Supplement: Supplementary file 1 [file nutrients-11-01930-s001.pdf]

Table S1. Characteristics of the participants included and excluded from the analysis, OsteoLaus study, Lausanne, Switzerland.

	**Included**	**Excluded**	***p*-Value**
Number	839	214	
Age (years)	62.8 ± 7.5	64.2 ± 8.0	0.014
Educational level (%)			<0.001
University	141 (16.8)	21 (9.8)	
High school	220 (26.2)	50 (23.4)	
Apprenticeship	332 (39.6)	73 (34.1)	
Mandatory	146 (17.4)	70 (32.7)	
Smoking categories (%)			0.434
Never	367 (43.7)	91 (45.3)	
Former	321 (38.3)	68 (33.8)	
Current	151 (18.0)	42 (20.9)	
Body mass index (kg/m^2)	25.4 ± 4.1	26.3 ± 4.7	0.004
BMI categories (%)			0.038
Normal	414 (49.3)	89 (41.6)	
Overweight	305 (36.4)	81 (37.9)	
Obese	120 (14.3)	44 (20.6)	
Sedentary status (%)	543 (64.7)	44 (71.0)	0.319
Diabetes (%)	40 (4.8)	24 (11.3)	<0.001

BMI, body mass index. Results are expressed as a number of participants (percentage) for categorical variables and as average ± standard deviation for continuous variables. Between-group comparisons performed using chi-square for categorical variables and student's t-test for continuous variables. Percentages in the excluded group might not correspond to the total sample size, due to missing values.
